# Vitamin C Acutely Affects Brain Perfusion and Mastication-Induced Perfusion Asymmetry in the Principal Trigeminal Nucleus

**DOI:** 10.3389/fnsys.2021.641121

**Published:** 2021-02-26

**Authors:** Andrea Viggiano, Sara Ponticorvo, Antonietta Canna, Carmine Secondulfo, Ludovico Sbordone, Antonio Russo, Marcellino Monda, Francesco Di Salle, Fabrizio Esposito

**Affiliations:** ^1^Department of Medicine, Surgery and Dentistry “Scuola Medica Salernitana”, University of Salerno, Baronissi, Italy; ^2^University Hospital “San Giovanni di Dio e Ruggi D’Aragona”, Salerno, Italy; ^3^Department of Advanced Medical and Surgical Sciences, University of Campania “Luigi Vanvitelli”, Naples, Italy; ^4^Section of Human Physiology, Unit of Dietetic and Sport Medicine, Department of Experimental Medicine, University of Campania “Luigi Vanvitelli”, Naples, Italy

**Keywords:** arterial spin labeling, magnetic resonance imaging, mastication, trigeminal nuclei, local brain perfusion, vitamin C (ascorbic acid)

## Abstract

Prolonged mastication may induce an asymmetric modification of the local perfusion of the trigeminal principal nucleus. The aim of the present study was to evaluate the possible influence of vitamin C (vit. C) on such effect. Four groups of healthy volunteers underwent arterial spin labeling magnetic resonance imaging (ASL-MRI) to evaluate the local perfusion of the trigeminal nuclei after a vit. C-enriched lunch or a control lunch. Two ASL-MRI scans were acquired, respectively, before and after a 1 h-long masticating exercise or a 1 h long resting period. The results showed (i) an increased global perfusion of the brain in the vit. C-enriched lunch groups, (ii) an increased local perfusion of the right principal trigeminal nucleus (Vp) due to mastication, and (iii) a reduction of the rightward asymmetry of the Vp perfusion, due to mastication, after the vit C-enriched meal compared to the control meal. These results confirmed a long-lasting effect of prolonged mastication on Vp perfusion and also suggest a possible effect of vit. C on cerebral vascular tone regulation. Moreover, the data strongly draw attention on the side-to-side relation in Vp perfusion as a possible physiological parameter to be considered to understand the origin of pathological conditions like migraine.

## Introduction

There is a growing body of evidence that genetic, epigenetic and environmental factors produce anatomical and functional brain asymmetries both in humans and animals (Ocklenburg and Güntürkün, 2018; Güntürkün et al., 2020). Handedness and hemisphere language specialization are just two of the most frequently observed, but other kinds of asymmetries are emerging and deserve attention. For instance, migraine, which is also a lateralized painful syndrome, is supposed to have a vascular origin, with possible anatomical (Khan et al., 2019) or functional asymmetries in brain perfusion (Andersen et al., 1988; Sanchez del Rio et al., 1999) with a particular relevance for brainstem perfusion (Afridi et al., 2005; Cucchiara et al., 2013; Russo et al., 2017, 2018; Zhang et al., 2018). Thus, the present study was focused on revealing possible physiological asymmetries in vascular tone regulation in human brain.

Many studies have demonstrated that the oxidative status affects the control of vascular tone (Pennathur and Heinecke, 2007; Förstermann, 2010). Indeed, it has been shown that an oral ingestion of vitamin C (vit. C) and E prevents the reduction of flow-mediated dilation that is observed after an oral glucose load (Title et al., 2000) or after prolonged sitting (Thosar et al., 2015). It has also been described that the intravenous injection of ascorbic acid has a direct vasodilation effect on the inoculated vessel (Grossmann et al., 2001) and that an oral ingestion of a fruits and vegetable purée-based drink, known to significantly rise plasma vitamin C, increases endothelium-dependent vasodilation (George et al., 2013). However, although it has been suggested that vit. C can have an effect on the nitric oxide-mechanism of vascular tone regulation, it is still questioned whether the vascular effects of vit. C can derive from scavenging of oxidant species (Trinity et al., 2016).

Recently it has also been reported that vit. C seems to have an opposite effect on cerebral vascular regulation; in fact, the acute increase in cerebral blood flow (CBF) or perfusion, induced by a single SCUBA dive with exposure to hyperoxia, was reduced in subjects taking a supplementation of vit. C for 6 days (Barak et al., 2018). In this context, a very intriguing observation was that the intravenous administration of vit. C markedly decreased fMRI signal changes during standard tasks (Di Salle et al., 1997). These observations raise the question on what effects dietary vit. C can have on CBF in everyday life.

It is noteworthy that regional CBF is continuously regulated by neuronal oxygen demand. This regulation can also have long-term effects due to prolonged activity of neurons; in particular, it has been demonstrated that a prolonged (1 h-long) mastication induces a long-term increase in local perfusion of the trigeminal principal nucleus with an asymmetric effect between right and left sides (Viggiano et al., 2015). It has also been noted that a prolonged painful stimulus, or a prolonged mastication overload, produces a long-lasting increase in the production of reactive oxygen species within the spinal trigeminal nuclei (Viggiano et al., 2005; Viggiano A. et al., 2010; Viggiano E. et al., 2010). Thus, it can be questioned whether vit. C can (i) acutely affect global brain perfusion and/or (ii) affect the levels or the asymmetry of local perfusion due to prolonged mastication.

The aim of the present study was to address these questions using arterial spin labeling (ASL) magnetic resonance imaging (MRI) in a human ASL-MRI study of prolonged mastication as previously described (Viggiano et al., 2015). The interest in this topic is strongly related to the interest in gaining knowledge of the physio-pathological mechanisms underlying migraine. The link between mastication, trigeminal nuclei and migraine is, in fact, suggested by two simple considerations: (i) the spinal trigeminal nuclei are involved in migraine pathophysiology (Mainero et al., 2011; Schulte and May, 2016), with an increased activity in response to nociceptive stimulation in the pre-ictal phase of migraine attacks (Stankewitz et al., 2011); (ii) chewing activity can be a trigger factor for migraine episodes (Lippi et al., 2015). The working hypothesis was that a prolonged mastication could induce a lateralized long-lasting increase in the trigeminal nuclei perfusion and that the oral ingestion of vit. C could eventually mitigate that effect.

## Materials and Methods

### Participants

Healthy volunteers (29 men, 10 women, aged 19–29 years) were enrolled for this study. The same sample size of a previous ASL-MRI study was chosen because the experimental design and the expected effect size were similar (Viggiano et al., 2015). All participants were right-handed except 3 men and 1 woman. Handedness was assessed by the question “Which hand do you use for handwriting?” Special attention was given to assess chewing-side preference (see below). Inclusion criteria were: (i) subjects were not affected by primary or secondary headache and had less than a few spontaneous non-throbbing headaches per year, no family history of migraine, no temporo-mandibular joint disorder, no congenital or functional hypertrophies, no emotional disorders (evaluated by a neurologist expert in headache medicine), (ii) subjects did not refer painful symptoms from any head region (e.g., tooth pain or any other oro-pharingeal pain, fever, or cold-like symptoms, etc.) during the last week.

The participants were pseudo-randomly divided in four groups (see section “Experimental Design”), with balanced distribution for sex, age, chewing side preference and handedness. The participants were also asked to avoid all possible sources of oxidants or antioxidants since the night preceding the day of the experiment, such as coffee, tea, chocolate, fruits, vegetables, packaged foods, spicy foods, mint, oil, smoking, and any kind of drug; in particular, on the day of the experiment, the participants were asked to have breakfast with white milk and some biscuits. Finally, they did not take anti-inflammatory drugs or pain relievers for any reason.

On the day of the experiment, before lunch, each participant underwent a chewing-side-preference test as previously described (Mc Donnell et al., 2004; Viggiano et al., 2015). Briefly, the participant was asked to chew a gum on his posterior teeth, being unaware of the scope of the test; after 15 s the position of the chewing gum was recorded as right or left, and the procedure was repeated every 5 s for 7 times. A preference score was then assigned for each side of the mouth counting the number of times the gum was found on that side. A particular chewing gum was kindly provided by Perfetti Van Melle Italia Srl (Lainate, Milan, Italy) having the same basic composition of commercial ones but void of any flavor or taste.

A formal approval to conduct the experiments was obtained from the internal review board of the University of Salerno (project identification code 31 dated 16/06/2015). All procedures fulfilled the national legislation and the Code of Ethical Principles for Medical Research Involving Human Subjects of the World Medical Association (Declaration of Helsinki). All participants gave their written informed consent prior to participate to the study.

### MRI Image Acquisition

Brain images were acquired using a 3 T MRI scanner (Siemens MAGNETOM Skyra, Siemens Healthcare, Erlangen, Germany), equipped with a head and neck 20 channel radio-frequency coil. The same imaging protocol was used for the two scans performed on each volunteer. Besides the initial localizer (three-planar scout), this included the following two sequences:

• 3D T1-weighted Magnetization Prepared RApid Gradient Echo (MPRAGE) sequence with repetition time (TR) = 2,400 ms, echo-time (TE) = 2.25 ms, resolution = 1 × 1 × 1 mm^3^, matrix size = 256 × 256, anterior-posterior phase encoding direction, generalized autocalibrating partially parallel acquisitions (GRAPPA) factor of 2 in phase-encoding direction, total acquisition time: 5.25 min. The MPRAGE scans were used for anatomical reference in the registration of the ASL scans.
• 3D Pulsed Arterial Spin Labeling (PASL) sequence with labeling scheme FAIR Q2TIPS, TR = 5,000 ms, TE = 16.38 ms, matrix size 64 × 64 voxels, voxel resolution = 3 × 3 × 3 mm^3^, bolus duration 700 ms, inversion time (TI) = 1,990 ms, 2 repetitions, 50 slices (total acquisition time: 5.25 min). This series was acquired with the subject at rest with eyes open.

### MRI Image Analysis

Single-subject whole-brain CBF maps were calculated from perfusion-weighted images (PWI; i.e., control-label differences from the PASL raw image series) according to the consensus formula in Alsop et al. (2015) which is implemented in-line in the MRI scanner software. Because no M0 image is acquired during the PASL sequence, a constant value for M0 is used in the formula and therefore the obtained CBF maps should be only considered semi-quantitative.

For the group-level analysis, the single-subject CBF maps were spatially normalized using the SPM12 toolbox^1^ running on MATLAB R2017a [The MathWorks, Inc., Natick, Massachusetts, United States, www.mathworks.com to the Montreal Neurological Institute (MNI) standard template (Evans et al., 1993)] using a three-step procedure: First, the control images from the 3D-PASL series were averaged to obtain an image with enhanced contrast, which was aligned in each subject to the corresponding anatomical 3D T1w image from the same session with an affine transformation. Then, all 3D T1w images from the first scan were segmented and normalized into a study specific template space with the non-linear diffeomorphic DARTEL approach (Ashburner, 2007) and then transformed to the standard MNI space. To ensure that the first and second ASL scans from each subject were normalized to exactly the same locations, the 3D T1-weighted of the second scan was registered to the 3D T1-weighted of the first session and the same transformation parameters (i.e., from the first scan) were used for normalization of both ASL scans. Thus, the initial affine transformation, the DARTEL estimated non-linear deformation fields and an isotropic 6 mm full width at half maximum (FWHM) Gaussian kernel were applied to the PWI maps of each subject and each scan.

**FIGURE 1:**
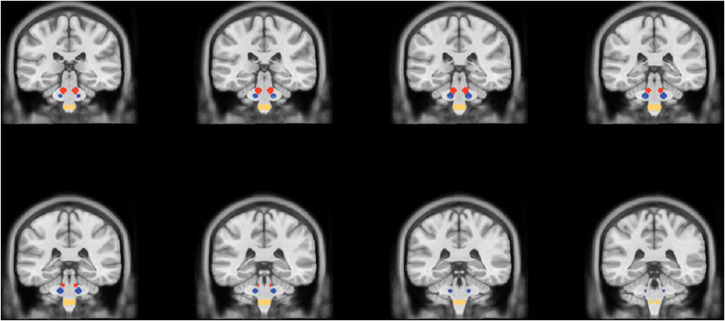
Coronal slices showing the location of the spherical regions of interest for the DM (red circles), the Vp (blue circles), and the Sp (yellow circles).

After spatial normalization, all individual CBF maps were imported in BrainVoyager (Brain Innovation, Maastricht, The Netherlands, www.brainvoyager.com) and further transformed to the Talairach space. In this space, the mean CBF over the entire brain mask was calculated as an estimate of the global perfusion in each subject and in each scan. Moreover, spherical regions of interests (radius = 5 mm) were created as previously described (Nash et al., 2009; Weigelt et al., 2010; Viggiano et al., 2015), for right and left principal trigeminal sensory nucleus (Vp; Talairach coordinates: ±12, 29, 31) and, as internal control, for the right and left dorsolateral region of the midbrain (DM, Talairach Coordinates: ±8, -27, -24) and for the right and left spinal trigeminal nucleus (Sp) pars oralis (Talairach coordinates: ±4, -29, -46) (Figure 1). The mean regional CBF was calculated from all voxels in each of these six ROIs (right and left Vp, right and left DM, right and left Sp) as an estimate of individual regional perfusion in each subject and in each scan.

### Experimental Design

Lunch took place between 12:30 and 13:00 and consisted of bread 80–90 g, with margarine for dressing; the lunch also included effervescent vit C (1 g) in a cup of drinking water (groups 1 and 2) or a cup of sparkling water (groups 3 and 4; Table 1).

**TABLE 1:** Synthesis of the experimental groups.

	Lunch with vit. C supplement	Control lunch
Resting for 1 h after the first ASL-MRI scan	Group 1	Group 3
Masticating for 1 h after the first ASL-MRI scan	Group 2	Group 4

One hour after lunch, a first ASL-MRI scan was acquired. Then the participants took a seat in a quiet room and contact electrodes were placed on the skin to record the electro-miography (EMG) from the masseter muscles. After placing the electrodes, the participants from groups 1 and 3 rested without chewing, while the participants from groups 2 and 4 were asked to chew a gum on one side of the mouth for 1 h maintaining a rate of at least 1 bite/s. The side for the experimental exercise was chosen pseudo-randomly, distributing homogenously subjects masticating on the preferred side (the side with the greater preference score) and subjects masticating on the opposite side; the same tasteless kind of gum described above was used to avoid any gustatory stimulation.

The EMG electrodes were connected to a computerized 2-channel electromyograph (I-330-C2+, J&J Engineering, Poulsbo, WA, United States) and a custom software written with LabView (National Instruments, Austin, TX, United States) provided an acoustic alert if no bites were sensed for more than 1 s. This ensured that all participants maintained the approximate rate of (at least) 1 bite/s for the entire exercise.

After the mastication or resting period, a second ASL-MRI scan was acquired.

### Statistical Analysis

The following variables were evaluated for each participant: (1) the perfusion value of the entire brain; (2) the local (regional) perfusion values of the ROIs for the trigeminal nuclei (right/left Vp, DM and Sp); (3) the normalized perfusion values, defined as the ratio of the regional to the global perfusion values from the same scan; (4) the regional perfusion change laterality index (LI) in each nucleus, defined as LI = (R - L)/(R + L), R and L being the ratio of the local perfusion values from the second scan to the first scan for the right (R) or left (L) nucleus.

All perfusion data were tested for Gaussian distribution by the Shapiro-Wilk test. To statistically test the presence and laterality of vit. C and mastication effects on global and local perfusion levels, global and local CBF data were separately entered into a multi-way analysis of variance (ANOVA) with repeated measures on nucleus (Vp/DM/Sp), time (basal/1 h) and, where applicable, side (right/left). LI data were entered into a two-way ANOVA with single measures. For all ANOVA models, vit. C (vs. water) and mastication (vs. rest) were specified as between-subject factors with two levels in such a way to account for all differences among the four experimental groups (water-rest, water-mastication, vit C-rest, vit C-mastication). These analyses were performed with JASP (Love et al., 2019)^2^. Pair-wise comparisons were performed with Student’s *t*-tests with Bonferroni correction or with the Tukey’s *post hoc* tests.

## Results

Most masticating participants only reported a mild muscular fatigue at the end of the mastication session, but nobody reported pain symptoms. The distribution of the chewing side score among al volunteers is shown in Table 2; due to pseudo-random assignment to the groups, there was no statistically significant difference between groups for this parameter.

**TABLE 2:** Distribution of the number of subjects for the chewing side preference score.

Chewing score		Subjects number
Right	Left	
0	7	2
1	6	1
2	5	9
3	4	14
4	3	4
5	2	4
6	1	2
7	0	3

All perfusion data showed a Gaussian distribution; the Shapiro-Wilk test did not report a significant difference from normally distributed populations.

**FIGURE 2:**
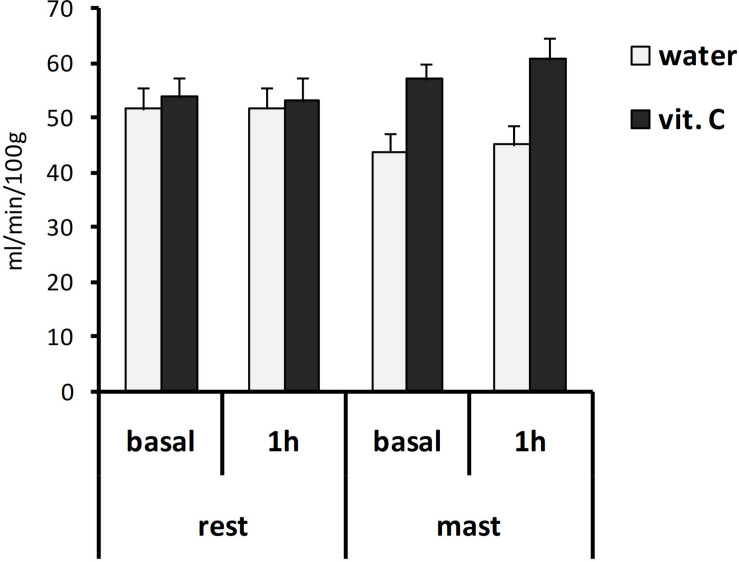
Global brain perfusion values. Bars represent the mean values obtained from the first (basal) or second (1 h) ASL-MRI scan, after a vit. C supplemented (vit. C) or control (water) lunch; the second scan was acquired after resting (rest) or masticating (mast) for 1 h. There was a significant effect for the water/vit. C factor (*p* < 0.05).

Global brain perfusion levels did not change significantly during the experiment (i.e., between the two scans), regardless of whether 1 h of mastication or rest was requested to the participants. However, these levels were found significantly higher in vit. C groups (56.3 ± 1.7 ml/min/100 g) compared to water-groups (47.3 ± 1.8 ml/min/100 g). The ANOVA with repeated measures on the time (basal/1 h) variable and two between-subject factors (rest/mastication, water/vit. C) showed a significant effect for the vit. C factor [*F*(1,35) = 6.03, *p* < 0.05; partial η^2^ = 0.15], whereas no main or interaction effects involving the other factors (time and mastication) were significant (Figure 2).

**FIGURE 3:**
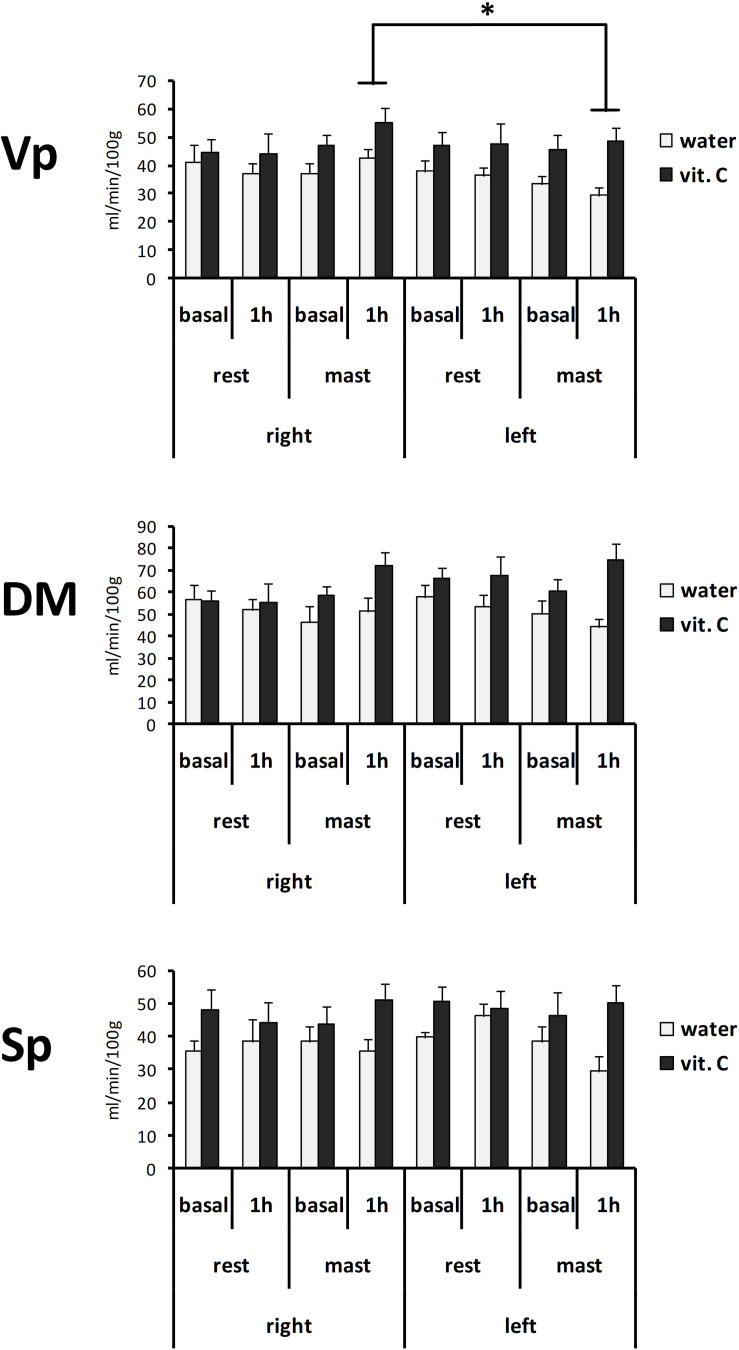
Perfusion values from the right or left side of the Vp, DM, and Sp trigeminal nuclei. Bars represent the mean values obtained from the first (basal) or second (1 h) ASL-MRI scan, after a vit. C supplemented (vit. C) or control (water) lunch; the second scan was acquired after resting (rest) or masticating (mast) for 1 h. There was a significant effect for the water/vit. C factor in all nuclei. ^∗^Tukey post hoc test, *p* < 0.05.

Local perfusion levels in the trigeminal nuclei of the vit. C-groups also showed higher values compared to the water-groups (Figure 3). The ANOVA with repeated measures on the nucleus (Vp/DM/Sp), time (basal/1 h) and side (right/left) variables and two between-subject factors (rest/mastication, water/vit. C) showed a significant effect for the factors nucleus [*F*(2,70) = 51.41, *p* < 0.01; partial η^2^ = 0.60], nucleus × side [*F*(2,70) = 6.87, *p* < 0.01], side x mastication [*F*(1,35) = 6.40, *p* < 0.05; partial η^2^ = 0.16] and vit. C [*F*(1,35) = 8.09, *p* < 0.01]. The Tukey’s *post hoc* test showed that in the masticating groups, irrespective of the water/vit. C factor, the perfusion within the Vp after 1 h was greater on the right side (49.33 ± 3.54 ml/min/100 g) than on the left side (39.97 ± 3.46 ml/min/100 g).

**FIGURE 4:**
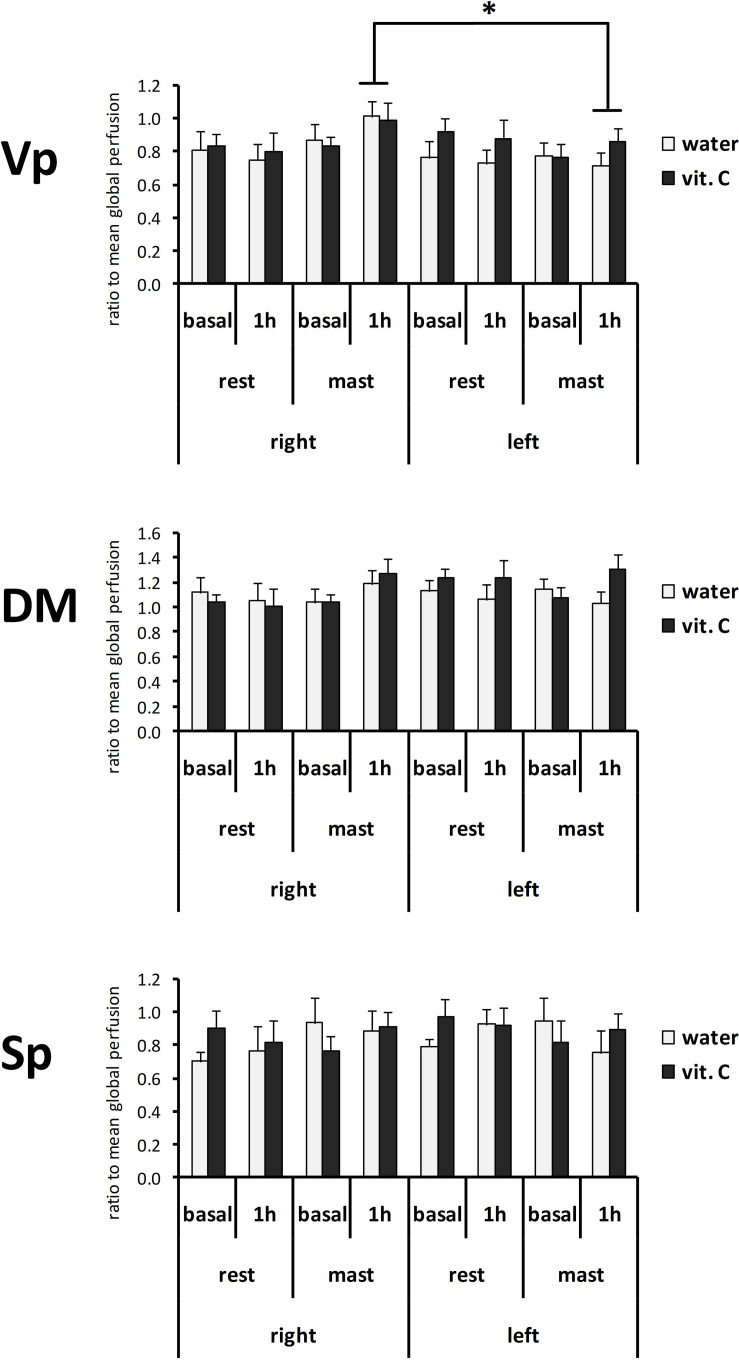
Normalized perfusion values from the right or left side of the Vp, DM, and Sp nuclei at first (basal) or second (1 h) ASL-MRI scan, after a vit. C supplemented (vit. C) or control (water) lunch; the second scan was acquired after resting (rest) or masticating (mast) for 1 h. Bars represent the mean ratio between the perfusion values and the global perfusion values obtained from the same scan. There was no significant effect for the water/vit. C factor. *Tukey *post hoc* test, *p* < 0.05.

The normalized perfusion values showed a significant effect for mastication in the Vp (Figure 4). The ANOVA with repeated measures on the nucleus (Vp/DM/Sp), time (basal/1 h) and side (right/left) variables and two between-subject factors (rest/mastication, water/vit. C) showed a significant effect for the factors nucleus [*F*(2,70) = 46.57, *p* < 0.01; partial η^2^ = 0.57], nucleus x side [*F*(2,70) = 7.57, *p* < 0.01; partial η^2^ = 0.18] and side × mastication [*F*(1,35) = 6.34, *p* < 0.05; partial η^2^ = 0.15], but not for the treatment factor (water/vit. C). The Tukey’s *post hoc* test showed that in the masticating groups, irrespective of the water/vit. C factor, the normalized perfusion values within the Vp after 1 h was greater on the right side (0.94 ± 0.06) than on the left side (0.76 ± 0.05).

**FIGURE 5:**
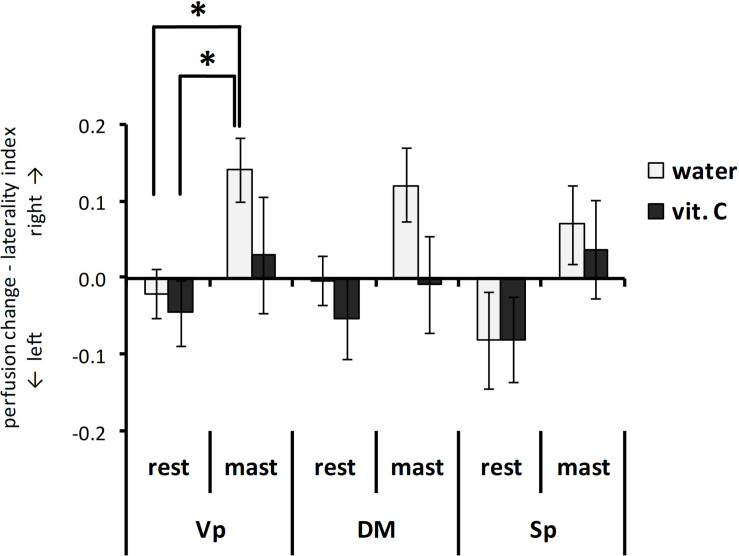
Perfusion change—laterality index. Bars represent the laterality index LI (see text for definition) obtained from each trigeminal nucleus after a vit. C supplemented (vit. C) or control (water) lunch and resting (rest) or masticating (mast) for 1 h. There was a significant effect for the mastication factor from the Vp and the Sp nuclei (*P* < 0.05). *t-test with Bonferroni correction for six multiple comparisons, *p* < 0.05.

The analysis of LI data highlighted a significant right side-prevalence of the perfusion change in the masticating groups compared to the resting groups (Figure 5). The ANOVA with repeated measures on the nucleus variable (Vp/DM/Sp) and with two between-subject factors (rest/mastication, water/vit. C) showed a significant effect for the masticating/resting factor [*F*(1,34) = 4.73, *p* < 0.05; partial η^2^ = 0.09]. From the series of *post hoc* pair-wise comparisons, there was a significant right-side prevalence of the Vp perfusion for the masticating-water group (LI = 0.14 ± 0.04) but not for the masticating-vit. C group (0.03 ± 0.08) compared to the resting groups (−0.02 ± 0.03 for the resting-water group, −0.04 ± 0.04 for the resting-vit. C group; Student’s *t*-test with Bonferroni correction for six multiple comparisons, *p* < 0.05).

All results were confirmed even after exclusion of left-handed participant. The effect on the laterality index in Vp, in particular, was still significant considering a single comparison (*p* < 0.012; Table 3).

**TABLE 3:** Results of the statistical analysis with or without exclusion of left-handed participants.

Experimental variable	Analyzed factors	All data			After exclusion of left-handed participants		
		Analysis	*p* <	Partial η^2^	Analysis	*p* <	Partial η^2^
**Global perfusion**	vit. C	ANOVA F1,35 = 6.026	0.019	0.147	ANOVA F1,32 = 5.482	0.026	0.146
**Raw perfusion values**	nucleus	ANOVA F2,70 = 51.406	0.001	0.595	ANOVA F2,64 = 57.183	0.001	0.641
	nucleus x side	ANOVA F2,70 = 6.873	0.002	0.164	ANOVA F2,64 = 5.552	0.006	0.148
	side x mastication	ANOVA F1,35 = 6.402	0.016	0.155	ANOVA F1,32 = 4.247	0.048	0.117
	vit. C	ANOVA F1,35 = 8.095	0.007	0.188	ANOVA F1,32 = 8.065	0.008	0.201
**Normalized perfusion values**	nucleus	ANOVA F2,70 = 46.572	0.010	0.571	ANOVA F2,64 = 54.229	0.001	0.629
	nucleus x side	ANOVA F2,70 = 7.568	0.001	0.178	ANOVA F2,64 = 5.835	0.005	0.154
	side x mastication	ANOVA F1,35 = 6.343	0.017	0.153	ANOVA F1,32 = 4.154	0.050	0.115
	side x time x mastication	ANOVA F1,35 = 4.421	0.043	0.112	ANOVA F1,32 = 4.874	0.035	0.132
**Laterality index**	mastication	ANOVA F1,34 = 4.729	0.037	0.093	ANOVA F1,32 = 5.432	0.026	0.145
	Vp mast.-water vs rest.-water	*t*-test	0.008		*t*-test	0.012	
		*t*-test-Bonferroni corr.	0.048		*t*-test-Bonferroni corr.	0.075	
	Vp mast.-water vs rest.- vit.C	*t*-test	0.004		*t*-test	0.008	
		*t*-test-Bonferroni corr.	0.024		*t*-test-Bonferroni corr.	0.048	

## Discussion

The present study demonstrated that local brain perfusion can be asymmetrically regulated in response to physical activity. This effect is likely the consequence of a functional asymmetry in neuronal activity and agrees with other literature data reporting asymmetrical reactivity of primary motor cortex (Driver et al., 2015). The present study demonstrated that the asymmetrical change in brain perfusion can persist after the physical activity (mastication) is stopped and enlarges the range of the possible causes and consequences of functional asymmetries. Future studies could verify, for instance, if a chronic functional asymmetry can eventually lead to a permanent, anatomical vascular asymmetry or if a transient but large asymmetry in perfusion could give rise to a painful syndrome (migraine). In this regard, it is worth nothing that an asymmetry in facial perfusion vascular pulsation has been demonstrated in migraineurs (Zaproudina et al., 2013) and that some anatomical asymmetry can also be found in migraineurs (Kaaro et al., 2008).

The data obtained in the present study demonstrated that an acute oral ingestion of vit. C can increase brain perfusion. Indeed, global perfusion levels observed in subjects from vit. C groups were, on average, significantly higher than those observed in subjects from control (water) groups. Such a baseline effect of vit. C on brain perfusion was actually unexpected and, to the best of our knowledge, is not described in current literature.

Contrary to the working hypothesis, the present data did not demonstrate a modulatory effect of vit. C on the mastication-induced local perfusion change in the trigeminal nuclei (Figures 3, 4). In previous studies, a relatively small increase of the local perfusion in the right Vp nucleus due to prolonged mastication (Viggiano et al., 2015) and an involvement of oxygen radical production within the Vp due to prolonged pain or mastication were shown (Viggiano et al., 2005; Viggiano A. et al., 2010; Viggiano E. et al., 2010). Thus, the ingestion of vit. C was initially supposed to be able to reduce the perfusion-change effect by oxygen radicals scavenging. The present data did not confirm this hypothesis, and it is possible that the direct (unexpected) effects of vit. C on global brain perfusion could overlap with the local effects of mastication resulting in a complex interaction, rather than a simple antagonist effect. Nevertheless, the results of the statistical analysis on perfusion data strongly drew attention on an effect on the side-to-side relation (Figure 5), which was already noted in the previous work (Viggiano et al., 2015). For this reason, the present data were also evaluated for the laterality of the perfusion change (i.e., between the right and left nuclei) using the LI index. By definition, LI intrinsically compensates the between-subject variability in the absolute (global or local) perfusion levels by taking the ratio between the two repeated scans (basal and 1 h scans). The LI analysis confirmed the right-side prevalence of mastication effects on the Vp nucleus. In this case, it was possible to show a statistically significant difference between the water-masticating group and the resting groups, while for the vit. C- masticating group, the LI index did not show a statistically significant difference from the resting groups. This result clearly shifts the relevance of the effect of prolonged mastication on the asymmetry in local perfusion levels between the two sides of the brain, as already suggested by previous findings (Viggiano et al., 2015). In addition, LI data suggest that vit. C may disrupt and, on average, clear or mask the disturbing effect on local perfusion symmetry between right and left Vp due to mastication, possibly by increasing the overall brain perfusion.

While the mechanism behind the impact of vit. C on brain perfusion is not clear, the physio-pathological relevance of the lateral asymmetry in brain perfusion is well documented in migraine literature. The simple fact that migraine is a painful syndrome preferentially affecting one side of the head, is evocative of a lateral dysfunction. MRI angiography shows changes in the circumference of the middle meningeal artery on the same side of pain (Khan et al., 2019). Moreover, it has been demonstrated that migraine is associated with lateralized regional changes in brain perfusion (Andersen et al., 1988; Sanchez del Rio et al., 1999) with a particular relevance of brainstem perfusion (Afridi et al., 2005; Cucchiara et al., 2013; Russo et al., 2017, 2018; Zhang et al., 2018). In the present study migraine and any other head pain syndrome were deliberately excluded to prevent that kind of bias. Thus, the present results indicate that physiological mechanisms can be responsible for the lateral asymmetry of local perfusion in the trigeminal nuclei, with a prominent involvement of the Vp, after a prolonged masticating activity. Such physiologic mechanisms could be the substrate for trigemino-vascular disorders, like migraine. In this frame, the clinical observations showing significant improvements on headache frequency and severity following the chronic administration (3 months) of vitamin C (150 mg/die) in migraine patients (Chayasirisobhon, 2013; Goschorska et al., 2020) may support the role played by vitamin C on brain structures involved in migraine pathophysiology such as the trigeminal nuclei. The main limits of the present study are the lack of a control scan before the ingestion of vit. C, the lack of a dose-response effect for vit. C administration. and the small sample size.

Due to the limited sample size, the effect on the laterality index in Vp could not be generalized to only right-handed peoples and further studies on larger samples are needed to evaluate the effect of different scores for handedness. In addition, the 3D-ASLvariant available at the time of the study (3D-PASL) did not allow the acquisition of a calibration image for the PWI signal resulting in semi-quantitative CBF maps. This might be the cause of a higher inter-subject and inter-scan variability in the CBF measurements. Finally, further studies on migraine populations can further elucidate the mechanisms underlying trigeminal functional abnormalities in migraine condition and in the ignition of attacks as well as the effects of Vit C on trigeminal nuclei in these patients. Chewing a chewing gum without taste for 1 h at a rate of at least 1 bite/s is not a common physiological condition, and the chewing gum is an ideal bolus. The chewing pattern and the muscular coordination between sides is influenced by the bolus hardness (Piancino et al., 2008) and the occlusion (Piancino et al., 2016). Thus the muscular activation during chewing is linked to the cranial structure and related force vectors and to the occlusion of teeth. These features could represent a predisposing factor in patients with migraine. The role of the neuro-muscular system must be considered at least as a co-factor. All these factors should be carefully considered in future studies to better characterize, and possibly explain, the acute and chronic pharmacodynamic and pharmacokinetic of vit. C effects on brain perfusion.

## Data Availability Statement

The raw data supporting the conclusions of this article will be made available by the authors, without undue reservation.

## Ethics Statement

A formal approval to conduct the experiments was obtained from the internal review board of the University of Salerno, project identification code 31 dated 16/06/2015. The patients/participants provided their written informed consent to participate in this study.

## Author Contributions

AV and FE: conceptualization, methodology, data curation, writing-original draft preparation, supervision, and funding acquisition. AV, FE, SP, and AC: formal analysis. SP, AC, and CS: investigation. LS, AR, MM, and FD: writing-review and editing. All authors have read and agreed to the published version of the manuscript.

## Conflict of Interest

The authors declare that the research was conducted in the absence of any commercial or financial relationships that could be construed as a potential conflict of interest.

## Footnotes

1 www.fil.ion.ucl.ac.uk/spm/

2 https://jasp-stats.org/
